# Investigation of blood biomarkers related to meat quality and quantity in Hanwoo steers

**DOI:** 10.5713/ajas.18.0191

**Published:** 2018-05-31

**Authors:** Yea Hwang Moon, Woong Ki Cho, Sung Sill Lee

**Affiliations:** 1 Department of Animal Science and Biotechnology, Gyeongnam National University of Science and Technology, Jinju 660-758, Korea; 2 Division of Applied Life Science (BK21 program) and Institute of Agriculture & Life Science (IALS), Gyeongsang National University, Jinju 52828, Korea

**Keywords:** Correlation, Blood Component, Carcass Traits, Retinol, Regression Equation, Hanwoo Steer

## Abstract

**Objective:**

This study was conducted to investigate the correlation between blood components and carcass traits, and to find the biomarkers related to meat quality and quantity in Hanwoo steers.

**Methods:**

One hundred twenty-six Hanwoo steers (8 to 9 months of age, body weight of 185.6±24.38 kg) were used to find the correlation between blood compositions and carcass traits. The steers were fed concentrate and rice straw (30 steers) or total mixed rations (96 steers) during the whole experimental period. Blood samples were collected from the jugular vein at the growing (8 to 12 months), fattening (13 to 23 months) and finishing phases (24 to 30 months). Steers were slaughtered at 30 to 31 months of age (body weight of 701.6±60.45 kg) and the carcass traits were evaluated. Blood metabolites and hormones were analyzed and the correlation coefficients and regression equations with carcass traits were determined.

**Results:**

Average concentrations of retinol, leptin and insulin were 1.10 IU, 30.34 ng, and 35.35 ng per mL of blood plasma, respectively. Retinol has negative correlations (p<0.01) with insulin and leptin. Blood insulin and total protein decreased with the age of steers, but retinol, aspartic acid transaminase (AST), glucose, cholesterol and triglyceride increased. In the finishing phase, significant (p<0.01) negative correlations occurred between blood retinol content and marbling score, and between blood AST content and *longissimus* muscle area of 13th rib, and the following regression equations were obtained: Marbling score (1–9) = −0.009×retinol (IU/100 mL)+ 9.125 (R^2^ = 0.643), *Longissimus* muscle area (cm^2^) = −0.250× AST(U/L)+112.498 (R^2^ = 0.450).

**Conclusion:**

It is possible to make highly marbled beef by controlling the blood retinol content during the fattening and finishing phases of Hanwoo steers. Accordingly, blood retinol and AST could be biomarkers for determining beef quality and quantity, respectively, prior to slaughter.

## INTRODUCTION

The beef industry is developing to meet consumer demand for higher quality production. So far, beef quality is associated with increasing the amount of intramuscular fat, or marbling. Because beef quality judgement depends exceedingly on the marbling score, the Korean government is trying to reform the beef quality criteria. Intramuscular fat, however, is still the most important index for beef quality in Korean food culture, which roasts meat directly over a charcoal fire.

Changes of blood components in ruminants have been used as a criterion of nutrient status [1] and as an index of metabolic disturbance [2]. Moreover, understanding the relationship between blood components and animal products might help to develop new feeding management. Feeding management for high quality beef with intramuscular fat deposit were used with castration, elongation of fattening period, and manipulations of dietary energy and vitamin A according to fattening phases. In Japan, many studies have been conducting to increase the quality of wagyu beef by manipulating the amount of dietary vitamin A during the fattening phase (Otani et al [3]) which was also focused on in the AFFRC [4]. Previous studies [5,6] reported that vitamin A has a negative (−) correlation with intramuscular fat synthesis in beef cattle. So, there have been attempts to produce highly marbled beef by inducing the deficiency of vitamin A during the fattening phase of beef cattle. We should, however, be careful about the manipulation of the feeding schedule, because vitamin A plays major role in growth and development, for the maintenance of the immune system and good vision. In Korea, some studies [7,8] on improving the quality of Hanwoo beef by manipulating the amount of dietary vitamin A have been conducted, but there have not been discussions on the relationship between blood retinol and beef quality. Accordingly, this study was conducted to investigate the correlation between blood components, including hormones, related to body fat deposition and carcass traits of Hanwoo cattle.

## MATERIALS AND METHODS

### Animals and feeding

One hundred twenty-six Hanwoo steers aged 8 months (initial body wt., 185.6±24.4 kg, housed with five calves per pen) were fed formula feed with rice straw or total mixed rations until 30 months of age (final body weight, 701.6±60.5 kg). The experimental period was divided into growing (8 to 12 months of age), fattening (13 to 23 months of age) and finishing phases (24 to 30 months of age).

Thirty steers were fed formula feed twice daily (at 0900 and 1700) at a body weight (BW) of 1.8%, 1.5%, and *ad libitum* during the growing phase, fattening phase and finishing phase, respectively, and fed rice straw freely.

Ninety-six steers were fed total mixed rations according to feeding programs. All the steers were allowed free access to water and mineral blocks. The steers were allotted to pens (5 m ×10 m, five animals/pen) with feeders divided by stanchion installed and were weighed monthly and feed intake was recorded weekly per pen.

The animal handling procedures of the present study followed the guidelines of the institutional animal care and use committee.

### Sample preparation and carcass trait assessment

Blood samples were collected at the end of the growing, fattening and finishing phases from the jugular vein at 3 h after morning feeding (1100 h). Blood plasma was obtained by centrifugation for 20 min at 3,000 rpm and stored at −70°C until analyzed. Carcass samples were harvested from steers (30 to 31 months of age) slaughtered at a commercial meat abattoir after fasting for 24 h. Carcasses were chilled for 24 h at 4°C, after which the left side was opened between the 13th rib and first lumbar and the *longissimus dorsi* (rib eye) muscle was then taken and used for grading the yield and quality of the carcass according to the standard criteria guided by [9].

A meat yield grade index (Y) was determined as 1, 2, 3 when the results of the following equation were below 63.3, 63.3 to 67.5, and above 67.5, respectively.

Y=68.184-(0.625×back fat thickness)+(0.130×rib eye muscle area)-(0.024×carcass wt.)+3.23

Meat quality grade (1 for lowest to 5 for highest) was estimated by regarding meat color (1 for very light red to 9 for very dark red), fat color (1 for white to 9 for yellow), texture (1 for soft to 3 for rough), and maturity (1 for youthful to 9 for mature) based on the marbling score (1 for lowest to 9 for highest).

### Analysis of blood retinol, biochemical components and hormones

High-performance liquid chromatography (HPLC, PerkinElmer, Series 200, Waltham, MA, USA) was used for the analysis of retinol in the blood of Hanwoo steers according to the modified methods of the Vitamin Society of Japan [10]. Blood plasma (50 μL) was placed into amber tubes containing 50 μL of ethanol, and the tubes were vortexed for 10 seconds. Then, 300 μL of n-hexane was added to the tubes and vortexed for 60 seconds. The tubes were centrifuged at 3,000 rpm for 5 min, and then 250 μL of supernatant was transferred to other tubes. After that, 300 μL of n-hexane was added to the precipitate and vortexed. The precipitate sample was re-centrifuged at 3,000 rpm for 5 min to thoroughly extract the retinol. The supernatants isolated with these steps were put together for a total amount of 500 μL. The supernatant was evaporated under N_2_ injection using a rotary vacuum evaporator attached to a spider tube set, and the residue was dissolved in 50 μL of iso-propanol. For the quantification of plasma retinol, 20 μL of aliquot was injected into the HPLC operating with the following conditions (Brownlee Validated C18 column with 5 μm pore size and 4.6 mm×150 mm length; detector, UV/Vis; mobile phase, MeOH : acetonitrile = 1:9; flow rate 1.0 mL/min; running time, 30 min; column oven temp., 40°C) by duplication. A calibration curve was prepared using retinol standard (Sigma-Aldrich, St. Louis, MO, USA).

Blood biochemical components including aspartic acid transaminase (AST), alanine transaminase (ALT), blood urea nitrogen (BUN), glucose, total protein, albumin, cholesterol and triglyceride were analyzed with the procedure and corresponding kit supplied by the manufacturer using an Automatic Biochemical Analyzer (HI System, Technicon, Tarrytown, NY, USA). Plasma insulin and leptin were assayed by enzyme-immunoassay using a multi-species insulin RIA kit and multi-species leptin RIA kit (Linco Research, Inc., St. Charles, MO, USA), respectively. I^125^ radioactivity was monitored using a γ-counter (COBRATM II, Packard Bioscience, Meriden, CT, USA).

### Statistical analysis

The differences in blood component concentration according to each phase (growing, fattening, and finishing) were evaluated using analysis of variance (PROC general linear model from SAS: Cary, NC, USA). The significance of differences was tested at p<0.05 using Duncan’s multiple range test [11]. From the blood data of the fattening and finishing phases, except for the growing phase, correlation coefficients and regression equations between blood parameters and carcass traits were analyzed by Pearson’s method and induced by [11], respectively.

## RESULTS AND DISCUSSION

### Blood biochemical components and carcass traits

The concentrations of blood retinol, insulin, leptin and biochemical components according to growing, fattening and finishing phases in Hanwoo steers are shown in Table 1. With the elapse of the fattening period, blood retinol, AST, glucose and total cholesterol concentrations increased significantly (p<0.05), but the concentrations of insulin, total protein and albumin peaked in the growing phase and thereafter decreased to the finishing phase. The blood non-esterified fatty acid (NEFA) concentration was significantly lower (p<0.05) in the fattening phase than in the other phases.

AST is commonly measured clinically to determine liver health; however, the reason for increased AST may come from other tissues such as muscle [12]. In this study, high blood AST with total cholesterol and triglyceride in the finishing phase was caused by a burden on the liver and an accumulation of intramuscular fat from high concentrate feeding. Blood NEFA generated from lipolysis was relatively lower in the fattening phase when lipogenesis rapidly progressed [4].

The minimum, maximum and mean values of the carcass traits of steers fed concentrates with rice straw or total mixed rations until slaughter at 30 to 31 months of age are shown in Table 2.

The carcass characteristics of Hanwoo steers used in this study were higher in average marbling score and ribeye area, and similar in backfat thickness compared to those of KAPE [9].

### Correlation coefficients

The correlations among blood biochemical components, including hormones, are shown in Table 3. Blood retinol concentration had a negative correlation with insulin, but a positive relationship with AST, total cholesterol and triglyceride (p< 0.01). Insulin had a negative correlation with the concentrations of leptin, AST and glucose in blood (p<0.01). Leptin had a negative relationship with the concentrations of ALT and albumin, but a positive correlation with AST and total cholesterol in blood (p<0.01). Significant (p<0.01) positive correlations were found between AST and NEFA, and between ALT and BUN. BUN also had a positive correlation with total protein, albumin and NEFA in blood. ALT had a significant (p<0.01) negative correlation with glucose and total cholesterol in blood. Significant (p<0.01) positive correlations were also found between albumin and NEFA, between total cholesterol and triglyceride, and between triglyceride and NEFA, respectively. Blood compositions are regarded as an important index in judging suitable conditions for the health and production purpose of animals, and the correlation between blood compositions helps to understand nutrition and metabolism.

Ruminants are less sensitive to insulin than monogastric animals [13], since the secretion of insulin in ruminants is mainly stimulated by volatile fatty acids. In particular, n-butyrate and propionate strongly stimulate the secretion of insulin [14]. And a small amount of glucose is absorbed by the small intestine of ruminants caused by microbial fermentation in the rumen. Therefore, glucose utilization in the whole body of a ruminant is relatively dependent upon gluconeogenesis in the liver and other tissues [15]. This seems likely to be the reason for the negative relationship between blood glucose and insulin in this study. Insulin also had a negative relationship with leptin. Insulin acts as a potent secretagogue of leptin from adipocytes [16], but its action on leptin secretion in ruminants is still obscure [17]. Moslemipur et al [18] reported that circulating levels of insulin and leptin in blood are positively correlated, and with decreasing insulin and leptin levels, blood glucose, triglycerides, cholesterol, total protein, BUN and ketone body levels increased.

Finding blood composition bound up with meat quality and quantity in carcass traits would surely be important to make a proper feeding program for beef cattle production, since blood composition might be manipulated by feeding control. The correlation coefficients between blood biochemical components and carcass traits in Hanwoo steers are shown in Table 4.

Blood retinol concentration had a significant (p<0.01) negative correlation with marbling score and meat quality grade. Gorocica-Buenfil et al [19] reported that the depletion of vitamin A in the diet of beef cattle increased intramuscular fat and subsequently improved the quality of meat. Japanese Black cattle administered injectable vitamin A periodically during the finishing period had decreased (p<0.05) marbling scores compared with those that did not receive supplemental vitamin A [5]. Nade et al [20] fed cattle two different concentrations of oral vitamin A and reported that cattle fed at one-half the standard dose had increased marbling scores (p<0.05). In Angus-cross steers, marbling score and intramuscular fat content were higher (p<0.05) for early weaned steers fed no vitamin A than for high vitamin A treatments [21]. Gorocica-Buenfilet al [22] also reported that the marbling score for steers fed a diet supplemented with 2,200 IU of vitamin A per dietary kg was significantly lower than for those receiving no supplemental vitamin A. But Oka et al [5] concluded that supplemental vitamin A had no effect in cattle after 23 months of age because of maturing of adipocytes in intramuscular adipose tissue. Gorocica-Buenfil et al [23] reported that no differences in marbling or quality grade were detected between Angus-based steers (BW = 224 kg) fed either 0 or 2,200 IU of supplemental vitamin A per kg. In association with these results, Bryant et al [24] concluded that whatever the effect of supplemental vitamin A on marbling and quality grade, it might likely be affected by the flux of liver stores of vitamin A throughout the life of the animal and in relation to the maturing of the intramuscular adipose depot.

Blood leptin, which is secreted from adipocytes, had a positive correlation (p<0.05) with back fat thickness, whereas it had a negative correlation (p<0.01) with meat quality grade.

Geary et al [25] demonstrated that serum leptin level was significantly related to marbling score and back fat deposition in beef cattle. And Chung et al [26] reported that leptin showed a significantly (p<0.05) positive correlation with back fat thickness, whereas it had a negative correlation with carcass yield index in Hanwoo steers.

However, Yonekura et al [27] reported that serum leptin level in Japanese Black cattle was not associated with the marbling score of the *longissimus dorsi* muscle.

AST concentration had a negative correlation (p<0.01) with rib-eye muscle area, yield index, marbling score and meat quality grade.

Carcass weight had a negative relationship with total protein concentration, whereas it was positive with total cholesterol and triglyceride concentrations of blood. And total cholesterol concentration had a negative correlation with rib-eye muscle area, yield index, marbling score and meat quality grade (p< 0.01). But Otani et al [3] reported that Japanese Black steers in the mid-fattening stage with high total cholesterol and low vitamin A in blood had a significantly higher marbling score than those with high total cholesterol and high vitamin A and those with low total cholesterol and low vitamin.

Triglyceride concentration in blood was negatively correlated with marbling score and meat quality grade, whereas it was positive with meat yield grade (p<0.01). The triglyceride circulating in blood is derived from the mobilization of the deposited fat content [28]. In agreement with this study, Arnett [21] also reported that there was a negative correlation (r = −0.38) between serum fatty acid content and intramuscular fat percentage.

### Blood biomarkers related to quality and quantity of beef

In the components with a high correlation, we searched for blood biomarkers related to beef quality and quantity in Hanwoo production and obtained regression equations for estimating the quality and quantity of meat. The results of regression analysis based on the relationship between blood components and carcass traits are shown in Table 5.

Regarding the r square value of the equation, a rib-eye muscle area (cm^2^) for meat quantity and a marbling score (1 to 9) for meat quality could be estimated by the equations Y = 112.498–(0.250×AST, U/L) and Y = 9.125–(0.009×retinol, IU/100 mL), respectively. Moreover, blood AST might be a biomarker of not only the quantity but also the quality of Hanwoo beef production. Blood retinol, however, is affected by many factors including infection, inflammation and recent dietary intake, and some biomarkers are more sensitive to changes in liver vitamin A reserves than others [29].

Finally, we can conclude that it might be possible to make highly marbled beef by controlling blood retinol during the fattening and finishing phases of Hanwoo steers. Accordingly, blood retinol and AST could be biomarkers for determining beef quality and quantity prior to slaughter, respectively.

## Figures and Tables

**Table 1:** Concentrations of blood retinol, insulin, leptin and biochemical components during growing and fattening phases in Hanwoo steers

Items	Phases			Average

	Growing	Fattening	Finishing	
Retinol (IU/100 mL)	99.11±3.48^c^	104.96±2.26^b^	114.45±1.47^a^	110.27±1.20
Leptin (ng/mL)	25.56±1.53	32.15±3.00	31.17±1.95	30.34±1.37
Insulin (ng/mL)	49.37±8.46^a^	41.95±3.71^a^	28.11±2.39^b^	35.35±2.37
AST (U/L)	34.63±7.88^b^	43.86±7.14^b^	65.19±3.71^a^	54.71±3.24
ALT (U/L)	47.74±4.69	44.83±3.85	55.66±7.05	51.69±4.31
BUN (mg/100 mL)	78.44±11.34	64.61±9.90	52.89±8.74	60.2±6.00
Glucose (mg/100 mL)	45.48±7.05^b^	53.5±6.72^b^	73.99±3.59^a^	64.07±3.06
TP (g/100 mL)	14.51±1.27^a^	12.5±0.90^a^	9.48±0.54^b^	11.09±0.47
Albumin (g/100 mL)	4.71±0.31^a^	3.93±0.18^b^	4.05±0.16^b^	4.14±0.12
TCH (mg/mL)	90.66±13.57^b^	101.03±11.87^b^	150.29±7.26^a^	128.03±6.00
TG (mg/100mL)	7.70±1.08^b^	7.11±0.99^b^	11.51±0.50^a^	9.79±0.45
NEFA (μEq/L)	256.38±19.76^a^	140.61±8.77^b^	277.67±17.83^a^	223.67±12.41

AST, aspartic acid transaminase; ALT, alanine transaminase; BUN, blood urea-N; TP, total protein; TCH, total cholesterol; TG, triglyceride; NEFA, non-esterified fatty acid.

Mean±standard error.

a,b Means in the same row with different superscripts differ significantly (p<0.05).

**Table 2:** Carcass traits of Hanwoo steers fed a formula feed and rice straw or total mixed rations until 30 months of age

Items	No	Minimum	Maximum	Average	Standard deviation
Carcass weight (kg)	126	340.00	544.00	425.40	44.07
Back fat thickness (mm)	126	6.00	28.00	13.52	4.341
Rib-eye muscle area (cm^2^)	126	73.00	141.00	99.53	15.23
Meat yield index1)	126	57.84	72.23	65.69	3.62
Meat yield grade1)	126	1	3	1.71	0.61
Marbling score2)	126	2	9	6.71	2.06
Meat color2)	126	4	5	4.76	0.43
Fat color2)	126	2	3	2.88	0.33
Texture2)	126	1	2	1.11	0.31
Maturity2)	126	2	3	2.16	0.37
Meat quality grade2)	126	2	5	4.01	1.06

1) Meat yield index = 68.184–(0.625×Back fat thickness)+(0.130×Rib-eye area)–(0.024×Carcass weight)+3.23; Meat yield grade, low (1 point) to high (3 point).

2) Marbling score, low (1 point) to high (9 point); Meat color, very light red (1 point) to very dark red (9 point); Fat color, white (1 point) to yellow (9 point); Texture, soft (1 point) to rough (3 point); Maturity, youthful (1 point) to mature (9 point); Meat quality grade, low (1 point) to high (5 point).

**Table 3:** The correlation coefficients among blood biochemical components during fattening and finishing phases in Hanwoo steers

Items	Retinol	Insulin	Leptin	AST	ALT	BUN	Glucose	TP	Albumin	TCH	TG	NEFA
Retinol	1	-	-	-	-	-	-	-	-	-	-	-
Insulin	0.3130**	1	-	-	-	-	-	-	-	-	-	-
Leptin	0.1527	−0.2870**	1	-	-	-	-	-	-	-	-	-
AST	0.4500**	−0.6050**	0.5220**	1	-	-	-	-	-	-	-	-
ALT	−0.3160	0.4150**	−0.4550**	−0.6601*	1	-	-	-	-	-	-	-
BUN	−0.3680	0.5380	−0.5190	−0.8250*	0.8871**	1	-	-	-	-	-	-
Glucose	0.4161	−0.6161**	0.4490	0.9260	−0.6832**	−0.8261*	1	-	-	-	-	-
TP	−0.4042	0.6450	−0.5090	−0.8912	0.7391	0.9101**	−0.8912**	1	-	-	-	-
Albumin	−0.2954	0.4342	−0.4060**	−0.6341	0.7951	0.8720**	−0.6201**	0.7560	1	-	-	-
TCH	0.4921**	−0.5531	0.3950**	0.8151	−0.5560**	−0.6900	0.8312	−0.7841	−0.4862	1	-	-
TG	0.3230**	−0.3993*	0.1016	0.4351	−0.0368	−0.1398	0.4871	−0.3300*	0.0715	0.5290**	1	-
NEFA	0.1978	0.0769	−0.0500	0.5782**	0.4060	0.5051**	0.0697	0.5421	0.5191**	0.2536	0.4962**	1

AST, aspartic acid transaminase; ALT, alanine transaminase; BUN, blood urea-N; TP, total protein; TCH, total cholesterol; TG, triglyceride; NEFA, non-esterified fatty acid.

* p<0.05,

** p<0.01.

**Table 4:** The correlation coefficients between blood biochemical components during fattening and finishing phases and carcass traits in Hanwoo steers

Items	Carcass weight	Back fat thickness	Rib-eye muscle area	Yield index	Marbling score	Meat color	Fat color	Texture	Maturity	Meat quality grade	Meat yield grade
Retinol	0.1640	−0.0098	−0.2650**	−0.1850*	−0.4660**	0.1640	0.1900*	0.1123	0.0092	−0.3690**	0.2310**
Insulin	−0.1546	0.0725	0.4630	0.2430*	0.5380	−0.1591	−0.2420	−0.2020*	−0.0087	0.4920	−0.2010*
Leptin	0.1048	0.1800*	−0.3990*	−0.3830*	−0.3850*	0.1509	0.2760	0.1478	−0.0835	−0.3600**	0.0859
AST	0.2040*	0.1110	−0.6710**	−0.5020**	−0.7200**	0.3450	0.4740	0.2720	−0.0312	−0.6990**	0.3980
ALT	−0.1820*	−0.0734	0.4640	0.3560	0.5220	−0.3250	−0.4680	−0.1990*	−0.0245	0.5000	−0.2890
BUN	−0.2330*	−0.1040	0.5610	0.4460	0.6400	−0.3400	−0.4620	−0.2400	−0.0269	0.6250	−0.3480
Glucose	0.3010*	0.0516	−0.5703	−0.4330*	−0.6720	0.3780	0.4730	0.2690	0.0366	−0.6430	0.4510
TP	−0.2140**	−0.1155	0.6210*	0.4810	0.6960	−0.3910	−0.5190	−0.2670	0.0187	0.6770	−0.3870*
Albumin	−0.1512	−0.1371	0.4380	0.3810*	0.5060	−0.2730	−0.3650	−0.1960	0.0144	0.4810	−0.2870
TCH	0.3860**	0.0750	−0.4530**	−0.4110**	−0.6620**	0.3630	0.4320	0.1920	0.0777	−0.6570**	0.3470**
TG	0.2540**	−0.1279	−0.1650*	−0.0661	−0.3500**	0.1690	0.1571	0.0811	0.0859	−0.3560**	0.2920**
NEFA	−0.0616	0.0112	−0.0516	−0.0192	0.0182	0.0205	−0.0081	0.1241	0.0563	−0.2121	−0.0607

AST, aspartic acid transaminase; ALT, alanine transaminase; BUN, blood urea-N; TP, total protein; TCH, total cholesterol; TG, triglyceride; NEFA, non-esterified fatty acid.

* p<0.05,

** p<0.01.

**Table 5:** The regression equations (Y = a+bX) between blood components and carcass traits in Hanwoo steers

Dependent variables (Y)	Independent variables (X)	a	SE	b	SE	r^2^
Rib-eye muscle area	AST	112.498	1.538	−0.250	0.023	0.450
	TP	81.281	2.142	1.588	0.168	0.385
Marbling score	Retinol	9.125	0.102	−0.009	0.001	0.643
	AST	8.642	0.197	−0.037	0.003	0.518
	TCH	8.966	0.251	−0.018	0.002	0.438
	Insulin	5.239	0.225	0.039	0.005	0.325

SE, standard error; AST, aspartic acid transaminase; TP, total protein; TCH, total cholesterol.
